# First report of natural parasitism by *Telenomus remus* Nixon, 1937 on *Spodoptera latifascia* Walker, 1856 eggs in Puerto Rico: insights into spatial-temporal dynamics in a semiarid tropical region

**DOI:** 10.3389/finsc.2025.1727464

**Published:** 2026-01-26

**Authors:** Daiane Dalla Nora, Ricardo Rubin Balardin, Ana Paula G. S. Wengrat, Wesley Augusto C. Godoy, Jose Carlos Verle Rodrigues

**Affiliations:** 1 Department of Biology, University of Puerto Rico – Rio Piedras (UPR-RP), San Juan, Puerto Rico; 2 Department of Agronomy, Western Paraná State University (UNIOESTE), Marechal Cândido Rondon, Paraná, Brazil; 3 Department of Entomology and Acarology, “Luiz de Queiroz” College of Agriculture, University of São Paulo (ESALQ-USP), Piracicaba, São Paulo, Brazil; 4 Insect Management and Molecular Diagnostics Lab, Department of Agriculture - Animal and Plant Health Inspection Service USDAAPHIS-PPQ-S&T, Edinburg, TX, United States

**Keywords:** biological control, diversity, landscape, Noctuidae, parasitoids, Scelionidae, spatial distribution, wasp

## Abstract

*Telenomus remus* is one of the most effective parasitoids of eggs used to control key pests in agricultural systems. This wasp can parasitize many species within the genus *Spodoptera*, including *Spodoptera latifascia*. The abundance and effectiveness of *T. remus* are influenced by landscape composition, climatic factors, and host availability, which need to be better characterized. However, no studies have investigated how those factors simultaneously affect the population dynamics of *S. latifascia* and *T. remus* under field conditions. In this study, we used sex pheromone traps to investigate the dynamics of host-parasitoid interactions and the parasitism rate of *S. latifascia* eggs. We also examined how landscape structure and function influence host-parasitoid dynamics, as well as the impact of local weather on parasitoid and moth abundance and on oviposition behavior. Our results indicated that the type of pheromone used did not affect the attraction of either the parasitoid or the oviposition behavior of the host. The landscape metrics driving each insect species differed, while local weather variables showed similar effects on both species. Specifically, the total number of patches was positively related to the abundance of *T. remus*, whereas functional traits were closely related to the abundance of *S. latifascia*. Rising temperatures negatively affected egg and moth abundance, while high relative humidity was positively correlated with increased abundance of these species. This study presents, for the first time, an established population of *T. remus* in Puerto Rico, and insights into the temporal and spatial dynamics associated with *S. latifascia*.

## 1 Introduction

*Telenomus remus* Nixon, 1937 (Hymenoptera: Scelionidae) is an important egg parasitoid of many lepidopteran species. It has recently been reported in several countries in the Old World following the introduction of the invasive species *Spodoptera frugiperda* Smith, 1797 (Lepidoptera: Noctuidae) (1, 2). With its high parasitism rate, *T. remus* is a crucial tool in augmentative biological control, particularly against pests within the genus *Spodoptera* (3).

One of the earliest observations of the genus *Telenomus* in the Puerto Rican archipelago occurred between 1921 and 1922, when it was identified as a less common parasite of *Leucoptera caffeina* Washburn, 1897 (Lepidoptera: Lyonetiidae) (4). Later, in 1923, *Telenomus monilicornis* Ashmead, 1894 was observed in eggs of *Manduca sexta* Linnaeus, 1763 (Lepidoptera: Sphingidae) (4). In 1936, other *Telenomus flaviventris* Ashmead, 1896 was reported parasitizing the eggs of a sugarcane pest (4). Then, in 1944, an unspecified species of *Telenomus* sp. was collected on Mona Island, northeast of Puerto Rico (5). After that, no further reports of the genus *Telenomus* were found for Puerto Rico. On the other hand, other researchers have referenced a study on *T. remus* in Puerto Rico (6, 7). However, this research primarily focused on testing the host range of *T. remus* as a potential biological control agent for *S. frugiperda* in Florida, USA (8). The population of *T. remus* was first released in the Caribbean around 1976 as an innovative biological control method for *S. frugiperda* (9). To our knowledge, *T. remu*s has never been reported as an established population on the island of Puerto Rico.

Female *T. remus* can parasitize egg masses that possess defense mechanisms against natural enemies, such as multiple-layered scales and hairs commonly found on egg masses of many *Spodoptera* species (10, 11). This trait is crucial for their effectiveness in suppressing pests under field conditions. The *T. remus* parasitism rate is influenced by several factors, including host species, which has been documented among some species within the genus *Spodoptera* (12, 13). However, most available data were generated for a limited group in the genus *Spodoptera*, and the existing information was derived from laboratory conditions (12, 13). The parasitism rate by *T. remus* on *Spodoptera latifascia* Walker, 1856 has not been documented in the literature, as this species remains one of the least studied within the genus *Spodoptera*. There is a need for more research on other species to enhance the understanding of host-parasitoid interactions under natural field conditions.

*Spodoptera latifascia* can be found throughout Central America and the southern United States (14). The larval stage of this species is polyphagous, feeding on a wide variety of plant species (15). However, there is limited information in the literature about their actual impact on crops and population dynamics in the field. Trapping methods that utilize sex pheromones can facilitate moth sampling and enhance our understanding of their population dynamics. Sex pheromone is a type of semiochemical involved in intraspecific communication among animals (16). Unfortunately, there is no currently specific commercial sex pheromone available to use in trapping methodologies for *S. latifascia*. Pheromone trapping often captures non-target species, which is common with lures designed to attract noctuid moths (17). For this reason, exploring some alternative sex pheromones could be beneficial for studies investigating the spatiotemporal dynamics of *S. latifascia*.

Another intriguing behavior of *S. latifascia* is that females lack selectivity when choosing a host for oviposition, often laying hundreds of eggs across many plant species. Interestingly, they also appear to have an atypical oviposition pattern, depositing eggs on plants that may not support future larval development, including non-crop habitats (18). The imperfect relationship between oviposition preference and the developmental performance of offspring poses a challenge to the theory of plant-insect interactions (19). However, this behavior can play a crucial role in natural biological control, supporting key species of natural enemies, such as egg parasitoids, across different patches of the agricultural landscape. The number of eggs laid on other species of plants and at different locations can strategically sustain populations of egg parasitoids, such as *T. remus*.

Natural pest control is a vital ecosystem service provided by natural enemies, contributing significantly to food security (20). Many factors, including weather and changes in the environment, influence the effectiveness of natural pest control. Natural enemies locate their hosts using volatile compounds emitted by both plants and their hosts (21). The complexity of determining how pests find plant hosts and how parasitoids find their hosts is strongly related to the landscape (22).

The landscape can be described in terms of structure, function, and change (23, 24). The structure of a landscape refers to how its elements are arranged in space, including the layout, size, variety, and connection of patches, which are areas of land that stand out from their surroundings (23, 24). Function relates to how organisms interact with landscape elements, which can significantly influence how they perceive their environment when seeking suitable hosts. Changes in landscape structure and function occur over both space and time.

Agricultural landscapes typically exhibit low plant diversity, which alters the complexity of flora composition and significantly affects natural enemy communities (25). Large areas are dominated by a few crop species, reducing landscape heterogeneity and decreasing habitat diversity, which leads to a decline in natural pest control (26, 27). This decline destabilizes pest-natural enemy interactions, increasing the risk of pest outbreaks. No studies have explored the impact of the landscape on *S. latifascia* and the natural pest control provided by *T. remus*, such research is essential for strategic management to enhance natural pest control. Furthermore, *S. latifascia* does not exhibit a strong migration pattern and has limited flying capability, traveling only a few kilometers per generation (18). For this reason, this species could serve as a valuable indicator of the impact of local land cover changes on its population dynamics and on the associated egg parasitoids.

Incorporating studies regarding establishing populations of *T. remus* adapted to local conditions could significantly enhance the management strategies for controlling several important agricultural pests. In this paper, we discuss the alternative use of sex pheromone trapping, as well as the influence of agricultural landscape elements and local weather on the population dynamics of *T. remus* and *S. latifascia*. We aimed to investigate: (1) if the dynamics of host-parasitoid are affected by the type of sex pheromone over time, (2) the parasitism rate of *S. latifascia* eggs in field conditions, (3) the relationship between the landscape structure and function on host-parasitoids dynamics, and (4) the effect of the weather on the abundance of parasitoids, moths and oviposition behavior. This study contributes to the understanding of the influence of biotic and abiotic factors on the population dynamics of a host and its parasitoids under field conditions that have never been accessed before. Our results also demonstrated that *T. remus* is an efficient natural enemy.

## 2 Materials and methods

### 2.1 Study area

The study was conducted on a farm located in Guánica, Puerto Rico (17.981719, -66.897615). This area is situated in the subtropical dry zone along the southern region of the island. Guánica is characterized by extreme summer temperatures and prolonged periods of drought, which significantly affect agricultural practices, including the dynamics of pests and their natural enemies. The average annual temperature is around 27°C, with approximately 840 millimeters of rainfall per year (28).

This important crop-producing region is mainly covered by perennial crops throughout the year, with horticultural crops cultivated during the spring agricultural season. Farmers define the agricultural spring as the period from October to May when climatic conditions are optimal for crop development. During the hottest months of the year, the cultivated area decreases drastically due to higher temperatures and the onset of the hurricane season. Most of the land is overtaken by spontaneous vegetation between June and August, primarily invasive plant species.

### 2.2 Experimental designing and data collection

Our study area encompassed approximately 90 hectares (ha), representing the entire area of one farm. We assessed real-world conditions without changing the farmer’s management practices. This farm was selected for its highly diverse land cover composition throughout the year. Such diversification includes different species of crops and non-crop areas, which is crucial for studying the abundance of egg parasitoids and their hosts.

A unique oviposition behavior of female *S. latifascia* was studied to assess the parasitism rate on their eggs over time and across the local agricultural landscape. We observed that this species frequently laid eggs on plastic surfaces (PVC – polyvinyl chloride) in agricultural areas. This oviposition behavior is unexpected, as plastic is not an ideal environment for fragile neonates. That was first detected on green plastic traps (Bucket Funnel Trap, 20cm x 13cm), which are usually used to sample moths.

Sampling eggs to study natural parasitism poses challenges, primarily due to their greenish color, which makes them difficult to spot among vegetation. To address this issue, we used the oviposition pattern behavior of *S. latifascia* to aid our sampling efforts. This methodology proved effective at facilitating egg searches in the field, especially across different vegetation types. For this reason, we employed same sex pheromone traps to collect moths, eggs attached to the surface, and egg parasitoids.

**Figure 1:**
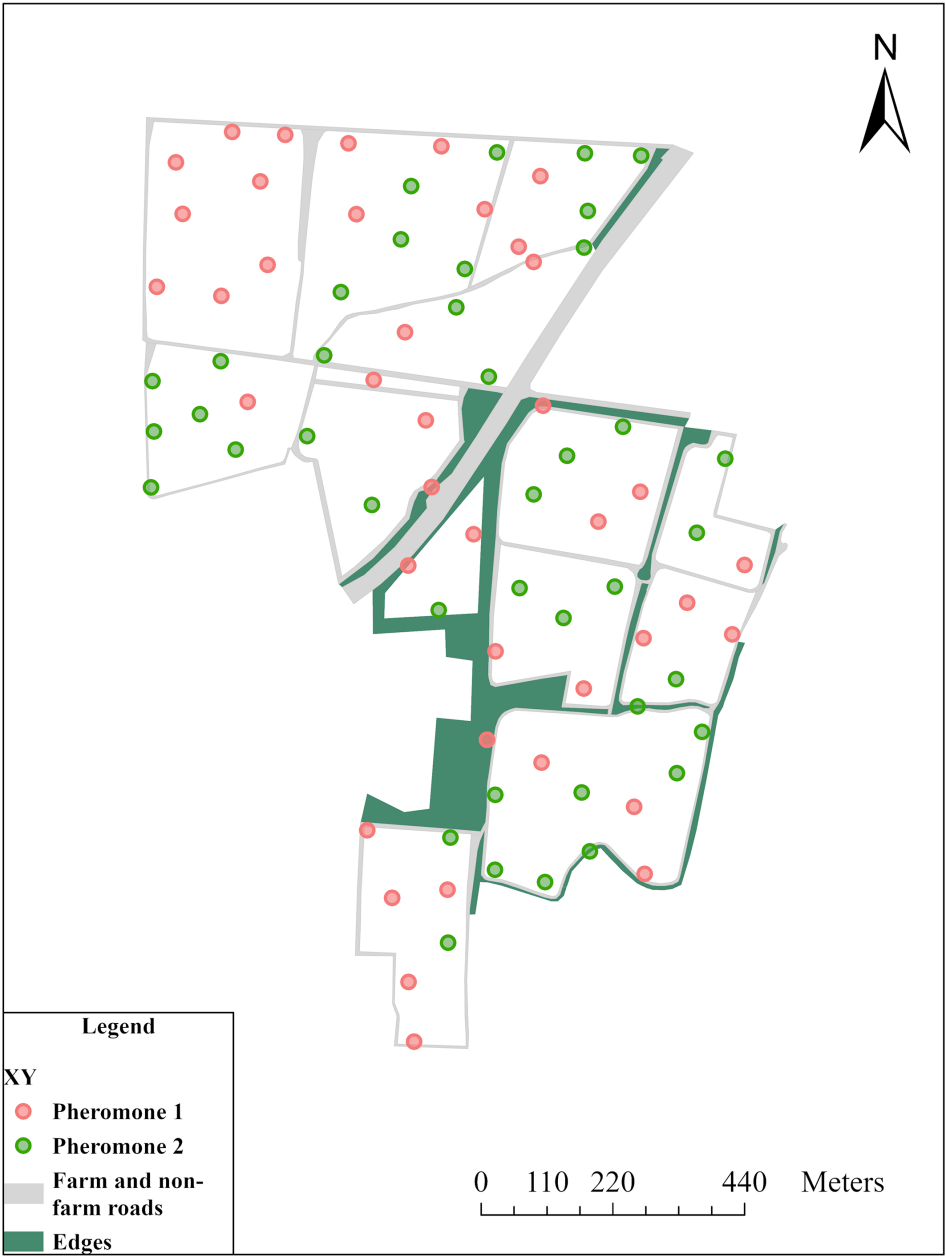
Map showing the study area in Guánica, Puerto Rico. The distribution of traps is indicated by red and green dots, labeled as Pheromone 1 and Pheromone 2, respectively. Pheromone traps were randomized and remained fixed throughout the field experiment. The green area represents the edges, primarily consisting of grasses, while the gray lines denote the roads in the region.

Pheromone trap points were established randomly, ensuring a minimum distance of 65 meters between each to minimize potential interference among the lures. All traps were positioned one meter above ground level. Randomization of trap placement and the establishment of minimum spacing between points on the sampling grid were conducted in ArcGIS PRO 3.4 software (ArcGIS Enterprise 11.4, Esri^®^). The georeferenced sample points were located using a handheld GPS (Global Positioning System) device (Gramin^®^). We used 80 traps, with 40 allocated two types of sex pheromone (1) (Z)-11-hexadecenal and (Z)-9-hexadecenal, and (2) (Z)-11-Hexadecenyl acetate, (Z)-7-Dodecenyl acetate, and (Z)-9-Tetradecenyl (Alpha Scents) (**Figure 1**). Lures were replaced every 14 days, coinciding with the collection of moths and eggs from the traps.

Data on the presence or absence of *S. latifascia* eggs on the trap and the moth abundance were collected from July 5, 2023, to July 24, 2024. The first detection of parasitoids in *S. latifascia* eggs occurred in December 2023, when wasps were observed parasitizing eggs on the surface of the traps. The study on the population dynamics of egg parasitoids started in January and continued until July 2024, every 14 days.

When eggs were found on the trap surface, we recorded the number of egg masses and carefully removed them with a brush, transferring them into 50 mL plastic containers. The samples were then transported to the laboratory, where they were kept under standard room conditions: 25 ± 2°C, 60 ± 9% RH, and 14:10 LD. After 10 days, each sample was analyzed using a stereoscope. We evaluated the total number of eggs, the number of hatched neonates, and the abundance of female and male parasitoids. The parasitoids were placed in a 1.5 mL centrifuge tube filled with 85% alcohol and stored at -20°C until morphological and molecular identification.

### 2.3 Parasitism rate

First, we calculated the prevalence of points where parasitoids were detected relative to the total number of sites with *S. latifascia* eggs observed on each sampling date. Second, to evaluate the number of parasitoids, we included only samples in which they were detected, as a large number of eggs were collected. When parasitoids were found in the egg samples, we counted the total number of eggs, neonates, and parasitoids. Finally, we calculated the percentage of parasitism based on the total number of eggs collected on each sampling date.

### 2.4 Laboratory *Spodoptera latifascia* rearing and morphological identification

The eggs were removed from the plastic surface of the pheromone traps and transferred to the laboratory. After hatching, the neonates were reared under artificial conditions. Larvae were fed on an artificial diet (General Purpose Lepidoptera F9772 - Frontier Agricultural Sciences) under controlled conditions, 25 ± 2°C, 60 ± 9% relative humidity (RH), and a photoperiod of 14 hours light and 10 hours darkness (14:10 LD) until they reached the adult stage. Adults and larvae were used for morphological identification (15, 29) (genitalia slide # 974, 975 S. Passoa collection).

### 2.5 *Telenomus remus* morphological and molecular identification

To analyze the surface morphology of the males and females of *T. remus*, scanning electron microscopy images were obtained using a high-resolution field-emission JEOL JSM-7500F SEM. Specimens were placed in sample holders and sputtered with a thin gold film (approximately 20 nm thick). The morphological terminology is based on Bin and Johnson (30)Johnson (31), and Talamas et al. (32).

Specimens of *T. remus* were sent to the Laboratory of Insect Biology (USP/ESALQ-Brazil) to perform morphological and molecular identification. 20 microscope slides were prepared primarily using the male genitalia, following the methods described by Polaszek and Kimani (33). In addition, 50 male specimens were prepared using Hoyer’s medium and mounted on additional slides. Morphological identification was based on the keys developed by Nixon (34) and Chou (35). Additionally, the specimens were morphologically and molecularly compared with a laboratory colony of *T. remus* maintained at USP/ESALQ since 2011. This colony originated from Venezuela, with parasitoids initially obtained from Trinidad and Tobago.

DNA extraction was performed using a nondestructive method described by Wengrat et al. (36). For each sampling date, DNA barcodes were obtained from six male and six female specimens. After DNA extraction and amplification, the cytochrome c oxidase subunit I (COX1) gene was amplified by polymerase chain reaction (PCR) using the procedures described by Gariepy et al. (37) and Wengrat et al. (36).

Bidirectional Sanger sequencing was performed at the Animal Biotechnology Laboratory of ESALQ. We verified, edited, and aligned the sequence chromatograms from each specimen using Geneious Prime 2022.1 software (https://www.geneious.com). The presence of NUMTs (nuclear parallels of mitochondrial origin) (38) was analyzed in MEGA X (39) following the methodology described by Corrêa et al. (40). Species confirmation was conducted by calculating the genetic distance utilizing the Kimura 2-parameter (K2P) model in MEGA X (39). The resulting sequences were deposited in the NCBI/BLASTn platform (www.ncbi.nlm.nih.gov), and the voucher specimens used for the molecular analyses were deposited in the Oscar Monte Entomophagous Insect Collection in the Instituto Biológico, Campinas, São Paulo, Brazil (IB-CBE 008802 to IB-CBE 008821). Specimens were also deposited in the National Museum of Natural History, Smithsonian Institution (USNM – 2097404, USNMENT01916843-USNMENT01916859) and in the Museo de Entomología y Biodiversidad Tropical of the University of Puerto Rico (MEBT-l 00488664).

A Maximum Likelihood phylogenetic tree was constructed using MEGA 12 software, with 1,000 standard bootstrap replicates using Nearest-Neighbor-Interchange method (Kumar et al., 2024). This phylogenetic tree incorporated eight sequences from seven countries: Brazil (MW834424), Suriname (GMSPA2987822), India (MN879316), South Africa (KMPUJ594519), and Benin (MK533751). We also included *Telenomus dilophonotae* from Brazil (OQ720989), *Telenomus nizwaensis* from Oman (MT635053), and *Hadronotus* sp. from Canada (JX968492) as outgroups.

### 2.6 Agricultural landscape classification and weather data

Irrigated agricultural landscapes, including our study area, follow a specific pattern in which crops are arranged based on the available irrigation system. This pattern allows the area to be divided into distinct patches (**Supplementary Video**).

In our study, we classified crop, non-crop patches, edge areas, roads, and forested areas based on field observations and satellite imagery and Earth Data Analytics, using ArcGIS PRO 3.4 software (Planet Labs^®^, Esri^®^). The land cover classification was conducted exclusively through on-site observations, on each sampling date, every 14 days, on the same day as the egg sampling. Classifying small areas with more details gives a more realistic quantification of habitat composition (41). We divided the landscape into structural and functional variables from January to July 2024 (**Table 1**).

**Table 1:** Structural and functional landscape categories were established to incorporate small habitats and measure heterogeneity over time.

Metrics	Categories	Description
Compositional and configurational heterogeneity	Patch Richness	Total count of distinct land cover types.
	Patch area	Total area (ha) of each type of crop and non-crop habitat.
	Green area	Total area (ha) covered with vegetation, excluding bare soil and mulching areas.
	Total number of structural patches	Total count of individual patches across all land cover types.
	Proportion of the largest patch^*^	Ratio of the largest patch divided by the total study area.
Functional^**^	Richness of flowering and non-flowering areas	Area of distinct land cover types, dividing each crop and non-crop habitat^***^ into non-flowering and flowering areas.
	Total flowering and non-flowering areas	Total area (ha) of each type of crop and non-crop habitat^***^, separated into non-flowering and flowering areas.
	Flower resources	Total area (ha) covered by crop flower resources.
	Total number of functional patches	Total number of land cover types, categorized into non-flowering and flowering areas.
	Proportion of flowering area	Ratio of the flowering and non-flowering area.

^*^This was used for the plantain and weed areas that represented the largest proportion of the farm during the study. High values indicate less fragmentation and low values suggest more fragmentation.

^**^The flowering resources were distinguished from vegetative areas.

^***^The monocrop area in this case is only areas composed of weeds.

We calculate the patch area for ten classes: coriander, cucumber, onion, plantain, pumpkin, tomato, watermelon, weeds, pepper, and bare land. We excluded boundaries, roads, and forest areas from the analysis since these features remain unchanged over time and space. The functional landscape analysis considered the total green area, flower availability, and crop residues. The total green area is a crucial category in Puerto Rican agriculture because farmers traditionally maintain their fields as bare soil during the fallow period. This practice can significantly impact the abundance of both the host and parasitoid species. Crop residues were areas that were no longer used but still contained the remains of the main crop.

Weather data were recorded by a weather station (Onset HOBO^®^ Data Loggers) placed in the study area (17.977564, -66.901918). Measurements of accumulated total precipitation (mm), relative humidity (%), and temperature (°C) were recorded every 30 minutes. For the analysis, we calculate the average values of relative humidity, temperature, and the accumulated precipitation for each sampling interval.

### 2.7 Data analysis

Because the data are not normally distributed according to the Shapiro-Wilk normality test (42), we used a Generalized Linear Model (GLM) that included a quadratic (second-degree polynomial – *X^2^*) term as a covariate. This method accounted for the nonlinear relationship between time and pheromones and the predicted probabilities of observing eggs, moths, and parasitoids. The model employed a binomial error and a logit link function, which converts probabilities into log odds ratios. The purpose was to identify differences in the attraction of these organisms over time, considering the type of pheromone used in the traps. A GLM with a gamma distribution and a log link function was used to analyze trends between the percentage of parasitism and the total number of eggs collected at each point using the MASS package (43). The pairwise comparison method was used to assess differences among pheromone levels using the emmeans package (44).

We also performed redundancy analysis (RDA) using the vegan package (45) to investigate the influence of structural and functional landscape metrics on the female oviposition behavior and the abundance of *S. latifascia* and *T. remus*. RDA is an alternative to canonical correlation analysis for exploring relationships between dependent and explanatory variables (46). We applied the Legendre method to minimize the impact of large abundance values between the three dependent variables (47). The functional analysis was divided into two models due to the high collinearity among the explanatory variables. To standardize the explanatory variables, we used the log (x+1) transformation. To retain the most important variables, we used the variance inflation factor (VIC < 10) to avoid multicollinearity (48), and then *R^2^* was calculated for each RDA model. A Monte Carlo permutation test with 999 interactions assessed the significance of the ordination. All analyses were performed with R software (version 4.4.2, 49).

To investigate how local weather affects *S. latifascia* oviposition behavior, moths, and *T. remus*, we employed GLM with a negative binomial error distribution and a log link function, using the MASS package (43). The negative binomial error was chosen because the variance exceeded the mean, indicating overdispersion in the abundance data. We also conducted residual analyses of the GLMs, which were verified using the DHARMa package (50). To visualize patterns in the distribution of egg and parasitoid abundance according to each type of land cover types, we created several spatial distribution maps using ArcGIS PRO 3.4 software (51).

## 3 Results

### 3.1 Morphological and molecular identification of *Telenomus remus*

**Figure 2:**
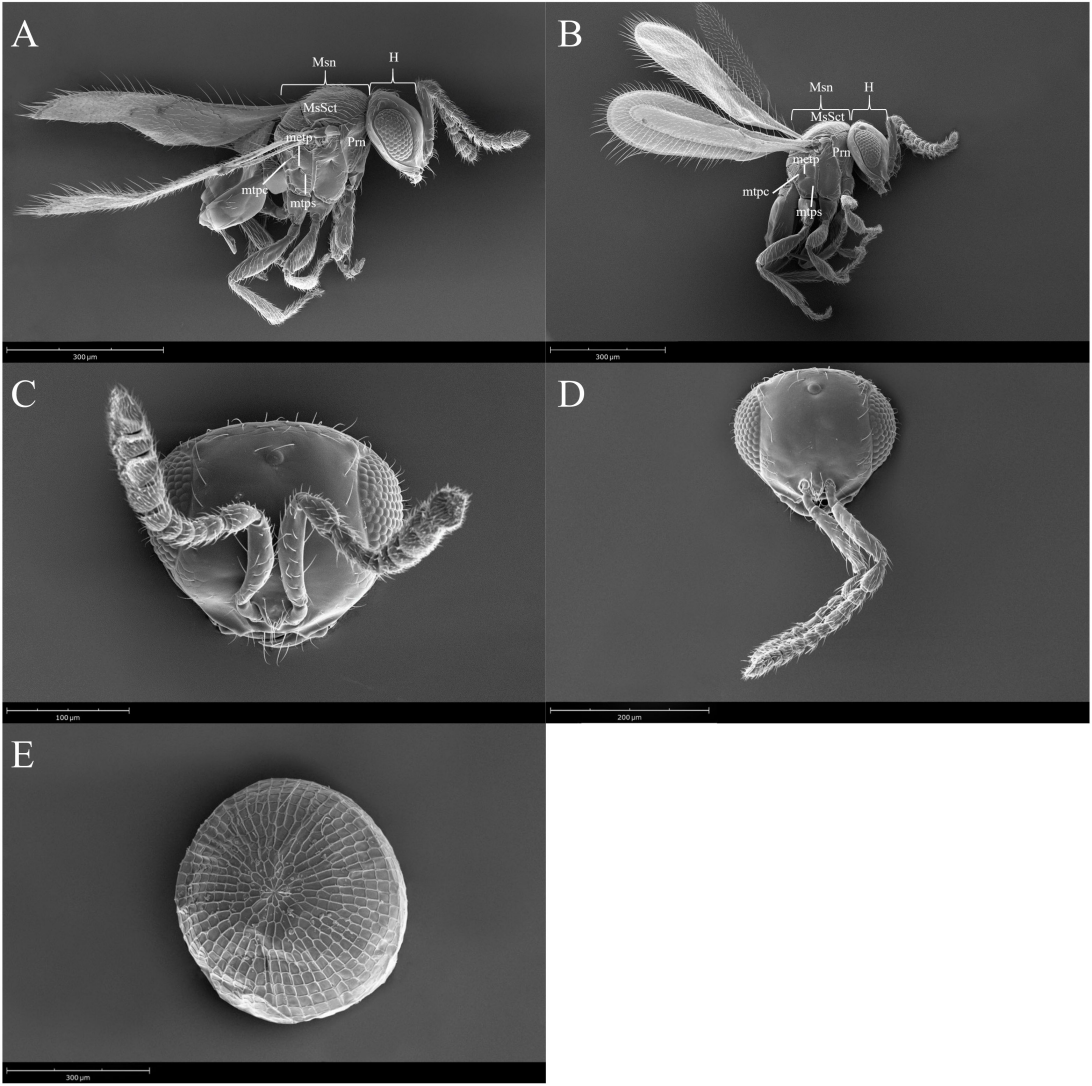
*Telenomus remus* images produced by scanning transmission electron microscope (STEM), the abbreviations in the figure indicate: mesonotum (Msn), pronotum (Prn), setae scattered on mesoscutum (MsSct), Pronotum (Prn), metapleural pit (Metp), metapleural carina (Mtpc), metapleural sulcus (mtps), and lateral view of the head (H). Female **(A)** lateral habitus, **(B)** male lateral habitus, female **(C)** head in frontal view, male **(D)** head in frontal view, and **(E)** top view of a *Spodoptera latifascia* egg.

All species of parasitoids were identified as *T. remus* (**Figure 2**) based on descriptions provided by Nixon (34) and Wengrat et al. (36). We successfully amplified a COI fragment of 556 base pairs. When we aligned these sequences with the Barcode of Life Data System and BLASTn, the results showed over 99% similarity with other *T. remus* sequences, confirming our species identification (**Supplementary Figure S1**). These sequences represent the first entries generated and deposited in the NCBI database for this parasitoid species from Puerto Rico, PV946711 to PV946723.

### 3.2 Pheromone preference and temporal dynamics

During the sampling period, we recorded the presence or absence of *S. latifascia* eggs and adults on 2,240 occasions. Of these, 590 points had eggs attached to the surface of the traps, while adults were trapped at 410 points over time. For pheromone 1, eggs were found at 288 points, and 195 moths were captured. In the case of pheromone 2, eggs were found at 302 points, and 215 moths were captured. Over the study period, *T. remus* was detected in 47 of the 291 sampling points that contained *S. latifascia* eggs. We collected 1,443 *T. remus* specimens from eggs attached to the surface of the traps, comprising 580 specimens from pheromone 1 and 863 specimens from pheromone 2.

**Figure 3:**
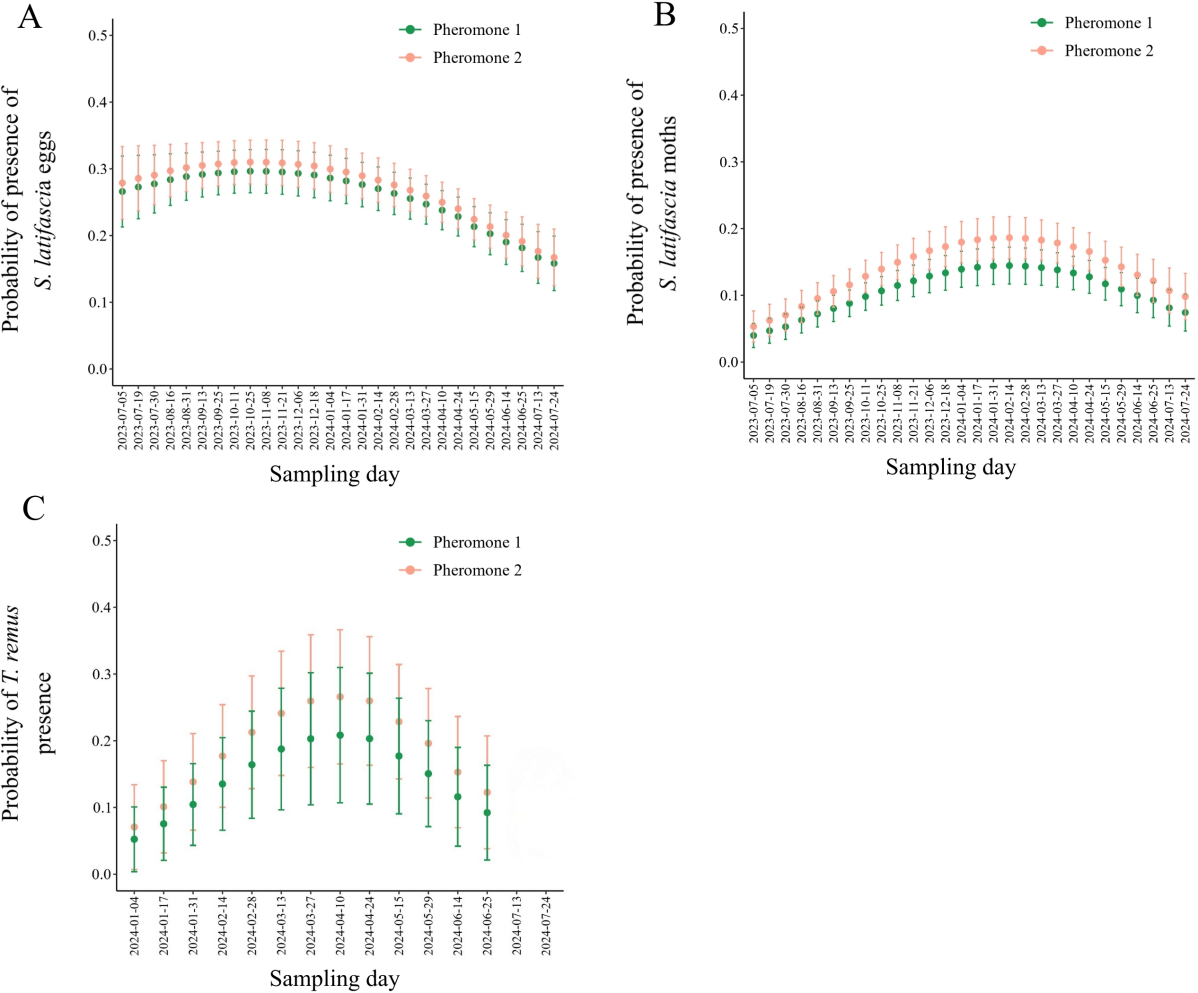
The relationship between time and the type of sex pheromone used in traps was analyzed based on the presence of **(A)***Spodoptera latifascia* eggs, **(B)***S. latifascia* moths, and **(C)***Telenomus remus.* Two types of pheromones were examined: (1) (Z)-11-hexadecenal and (Z)-9-hexadecenal, and (2) (Z)-11-Hexadecenyl acetate, (Z)-7-Dodecenyl acetate, and (Z)-9-Tetradecenyl. The dots on the graphs represent the adjusted values from a generalized linear model (GLM) that includes a quadratic term (*x²*) and assumes a binomial error distribution. The error bars indicate the 95% confidence intervals (CIs).

The model results assessing the effects of time and pheromone type show a decline in predicted probabilities of egg presence throughout the study (**Figure 3A**). There were no differences between the two pheromone types (Estimate = -0.065, SE = 0.96, *p*-value = 0.49). The first polynomial term (Estimate = -9.33, Std. Error = 2.37, *p*-value = 0.000^***^) and the second term (Estimate = -6.35, Std. Error = 2.34, *p*-value = 0.006^**^) both indicated a negative effect of time on the predicted likelihood of observing eggs attached to the traps. As expected, the relationship between time and the probability of finding eggs was non-linear. The intercept for pheromone type 1 suggested a negative effect on egg presence (Estimate = -1.07, Std. Error = 0.068, *p*-value = 0.000^***^), while pheromone 2 did not have a significant effect (Estimate = 0.065, Std. Error = 0.096, *p*-value = 0.49).

The predicted probability of *S. latifascia* presence increased from the beginning of sampling until May 2024, after which the population began to decline (**Figure 3B**). There was a statistical difference between the two pheromone types, with a higher odds ratio for pheromone 2 (Estimate = -0.305, SE = 0.13, *p*-value = 0.01^*^). The first term of the polynomial (Estimate = 9.30, Std. Error = 3.51, *p*-value = 0.008^**^) indicated a positive time effect, which did not accurately reflect the actual fluctuation of the population. In contrast, the second term (Estimate = -16.10, Std. Error = 3.43, *p*-value = 0.000^***^) provides a more accurate representation of the dataset, highlighting the negative effect of time. The predicted values for the probability of finding moths in the pheromone type were similar to the egg presence. For pheromone 1, there was a significant negative effect (Estimate = -2.13, Std. Error = 0.033, *p*-value = 0.000^***^), and pheromone 2 had a significant positive effect (Estimate = 0.30, Std. Error = 0.13, *p*-value = 0.019^*^).

The model that evaluated the overall effect of time on the predicted probability of *T. remus* presence on eggs indicated a decline in the parasitoid population over time (**Figure 3C**). There were no differences between the two types of pheromones (Estimate = -0.31, SE = 0.33, *p*-value = 0.34). Notably, the second-level polynomial covariate provides a better fit for the data (Estimate = -9.19, Std. Error = 3.31, *p*-value = 0.005^**^), demonstrating a decrease in the predicted probability of encountering parasitoids. Additionally, pheromone 1 had a negative effect on the predicted probability of parasitoid presence (Estimate = -1.99, Std. Error = 0.26, *p*-value = 0.000^***^). In contrast, pheromone 2 showed a positive effect, although not statistically significant (Estimate = 0.31, Std. Error = 0.33, *p*-value = 0.34).

Overall, the presence of both the host and the parasitoid decreased over the evaluated period. We observed no parasitoids associated with egg presence during the final two sampling dates (July 2024). This result could be attributed to abrupt changes in the composition of farmland cover.

### 3.3 *Telenomus remus* parasitism

When analyzing each sampling date as a unique unit, we found that the highest percentage of points with parasitoids was recorded in March and May, where 33.33% of the evaluated points exhibited parasitism (**Table 2**). Among the total number of eggs collected on each date, the highest parasitism level was observed in June, with 36.85% of the 521 eggs collected. When considering each point as a single unit, we recorded instances of 100% parasitism, with a total of 40 to 50 collected eggs. In other instances, parasitism rates varied between 85% and 95% at points containing 100 to 130 eggs.

**Table 2:** Summary of parasitism rate according to each sampling date. Guánica, Puerto Rico.

Date	Total n^o^. of egg masses	Points with egg presence	Points with parasitoids	% of points with parasitoids	Total number of neonates^*^	Total number of eggs**	*Telenomus remus* abundance^**^	Parasitism (%)^*^
2024-01-04	41	32	2	6.25	40	490	11	2.24
2024-01-17	76	30	5	16.67	125	539	50	9.28
2024-01-31	87	18	2	11.11	145	530	33	7.36
2024-02-14	39	19	2	10.53	11	366	34	9.29
2024-02-28	29	20	2	10.00	276	479	132	27.56
2024-03-13	21	15	5	33.33	21	370	64	17.30
2024-03-27	27	18	3	16.67	143	359	85	23.68
2024-04-10	29	15	3	20.00	319	463	140	30.24
2024-04-24	54	22	5	22.73	125	1151	156	13.55
2024-05-15	62	28	7	25.00	242	2965	233	7.86
2024-05-29	44	18	6	33.33	648	1651	292	17.69
2024-06-14	35	19	2	10.53	426	506	21	4.15
2024-06-25	36	21	3	14.29	329	521	192	36.85
2024-07-13	10	8	0	0.00	0	0	0	0
2024-07-24	18	8	0	0.00	0	0	0	0
Total	608	291	47	16.15	2850	10390	1443	-

*This parameter estimates only in the samples where parasitoids were detected.

^**^Many collected eggs did not hatch as neonates or parasitoids.

**Figure 4:**
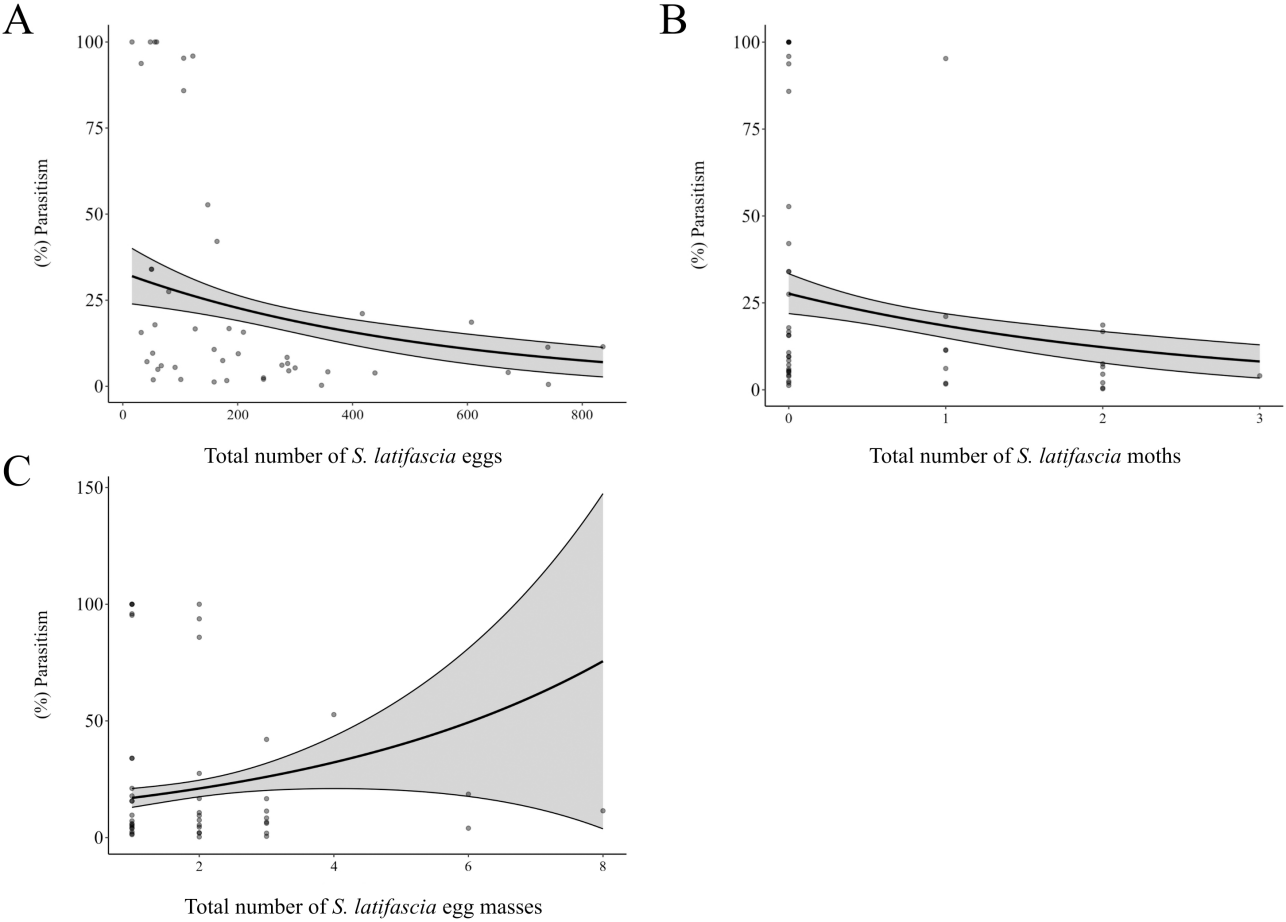
Percentage of parasitism according to **(A)** the number of *Spodoptera latifascia* eggs, **(B)** the number of *S. latifascia* moths, and **(C)** the number of *S. latifascia* egg masses at each dot. The solid line illustrates the predicted values from the generalized linear model (GLM) with a gamma distribution of the errors, and the shaded areas indicate 95% confidence intervals (CIs).

**Figure 5:**
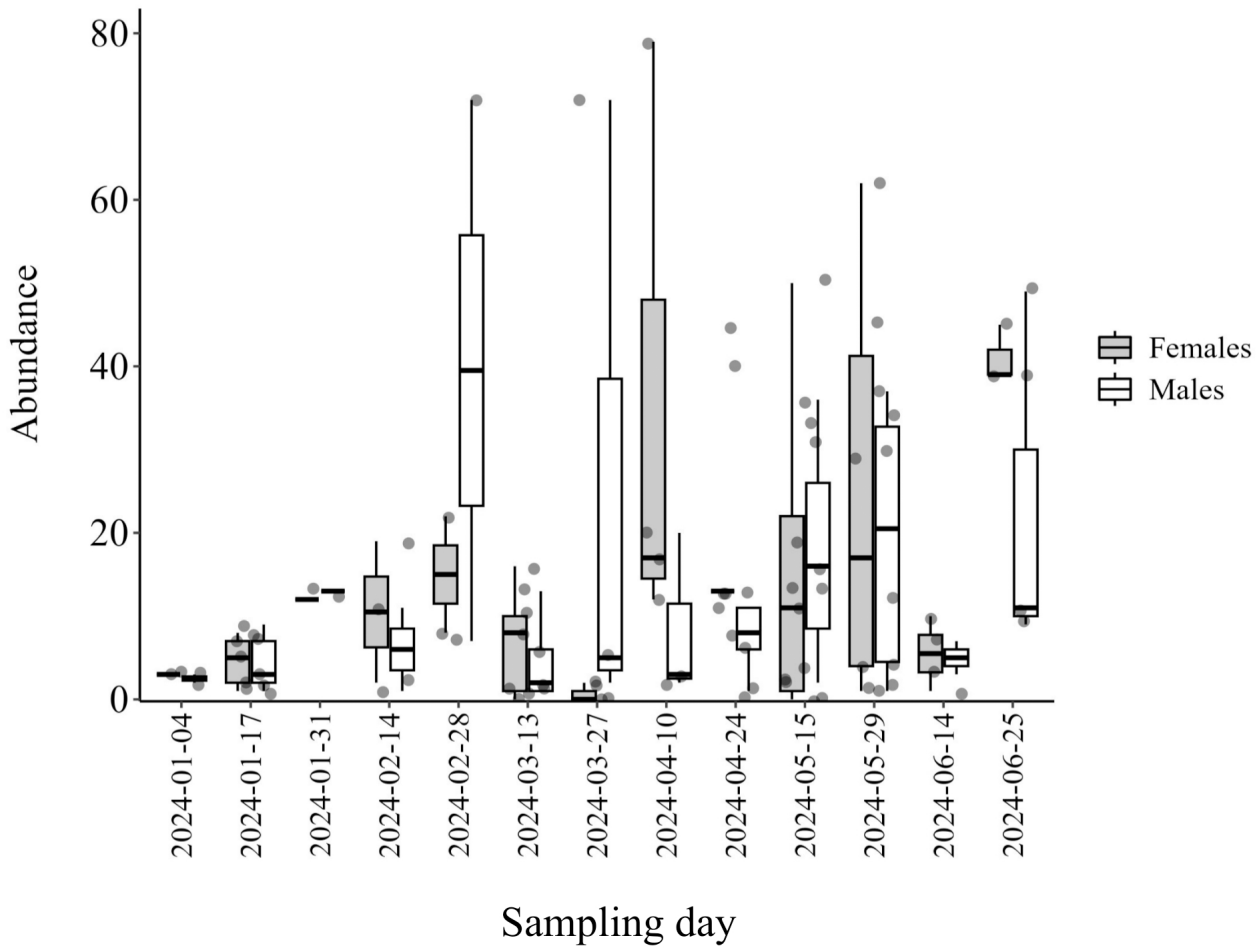
Distribution of the abundance of females and males of *Telenomus remus* over time. Guánica, Puerto Rico.

An increase in the total number of eggs per point indicates a decrease in the percentage of parasitism (Estimate = -0.002, Std. Error = 0.00, *p*-value = 0.01^*^) (**Figure 4**). The increase in the number of egg masses per point did not affect the percentage of parasitism (Estimate = 0.21, Std. Error = 0.15, *p*-value = 0.18). Additionally, the number of moths captured in the traps was negatively related to the percentage of parasitism (Estimate = -0.43, Std. Error = 0.24, *p*-value = 0.08). Besides that, the average parasitism rates and the proportion of males and females were calculated for each date (**Figure 5**, **Supplementary Table S1**). The highest sex ratio was recorded at the end of March, indicating a low number of female *T. remus* in the system.

### 3.4 Influence of land cover on the dynamics of *Spodoptera latifascia* oviposition behavior and moth abundance, and *Telenomus remus*

**Figure 6:**
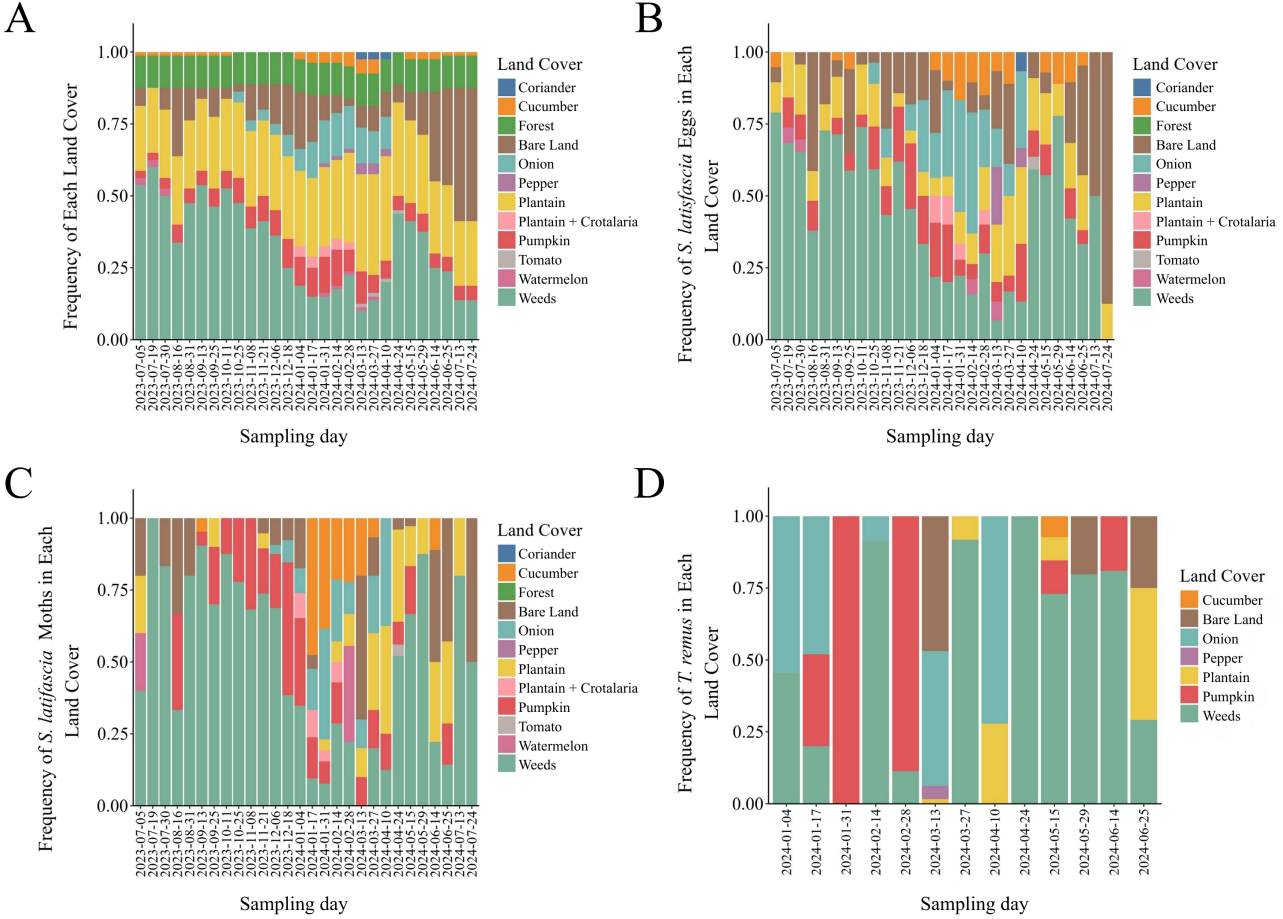
Temporal frequencies of each land cover were analyzed **(A)** across all points, only in the points where **(B)***Spodoptera latifascia* eggs, **(C)***S. latifascia* moths, and **(D)***Telenomus remus* were observed. Guánica, Puerto Rico.

During our study, we identified 12 distinct land cover types that constantly changed across space and time, affecting the population dynamics and interactions between *S. latifascia* and *T. remus*. Weeds, plantain, and areas of bare land made up more than 50% of the land cover observed at the points (**Figure 6**). We recorded a high frequency of eggs, moths, and parasitoids in weed-dominated areas and growing cucumbers, highlighting their critical role in the species dynamics.

**Figure 7:**
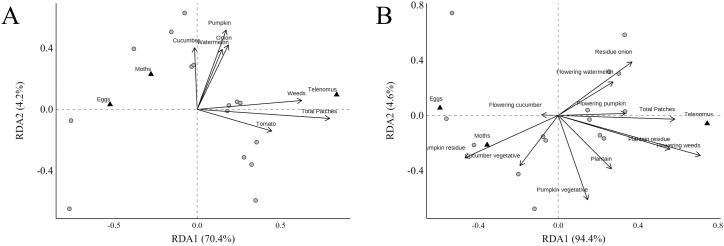
Redundancy analysis (RDA) was performed to generate biplots that illustrated the correlation between structural and functional landscape metrics and host-parasitoid dynamics. Only the most important explanatory variables were included in the analysis. The influence of **(A)** structural variables was indicated by an adjusted-*R²* of 0.50, while **(B)** functional factors had an adjusted-*R²* of 0.47.

The RDA ordination method illustrates the relationship between structural and functional landscape metrics and host-parasitoid dynamics (**Figure 7**). For the structural metrics, the two canonical axes explained 74.68% of the variance, with the first axis accounting for 70.46% and the second axis for 4.22% (**Figure 7A**). The abundance of points containing eggs and moths negatively affects the RDA1 axis. In contrast, the abundance of *T. remus* showed a positive correlation with the RDA1 axis, demonstrating a strong association with the number of patches and areas dominated by weeds (**Supplementary Table S2**).

In the functional analysis (**Figure 7B**), we examined the land cover traits that comprised the farm landscape in greater detail. The first axis accounted for 94.4% of the explained variance, while the second contributed 4.6%. These results indicate that the total functional number of patches, the area of pumpkin residues, and the area containing weeds in the flowering stage positively correlated with *T. remus*. Furthermore, areas with pumpkin residue and cucumber plants in the vegetative stage had a negative impact on *S. latifascia* moths. Flowering cucumber areas displayed a weak association with the number of points containing eggs. These findings suggest that land cover influences the dynamics of *S. latifascia* and *T. remus* differently. Additionally, the population dynamics of *S. latifascia* might be affected by other environmental metrics not assessed in this study.

Bare land cover could be a factor related to the absence of parasitoids in the *S. latifascia* eggs collected during the last two sampling dates (**Supplementary Video**). Additionally, we observed that traps surrounded solely by bare soil had the presence of eggs and parasitoids, indicating an imperfect plant-insect relationship and a key trait of the parasitoid, which is capable of parasitizing those eggs.

### 3.5 Influence of weather on the abundance of points with eggs, moths, and parasitoids

**Figure 8:**
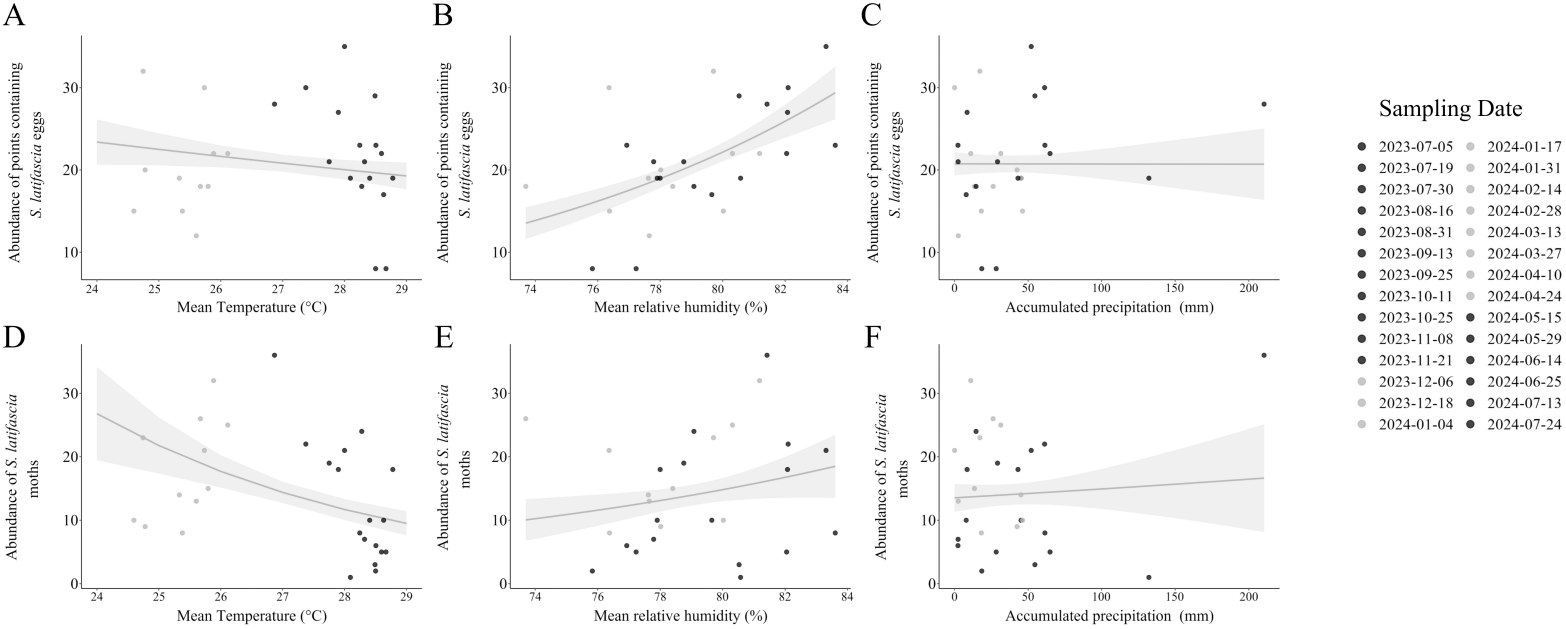
Effect of local weather on the abundance of points containing eggs, and the total number of moths of *Spodoptera latifascia*. The solid line illustrates the predicted values from the generalized linear model (GLM), and the shaded areas indicate 95% confidence intervals (CIs). The solid dots indicate the warmest time period and opaque dots the mildest time period as determined by temperature (°C) (**Supplementary Table S3**). The first model characterized the effects of the **(A)** mean temperature (p-value = 0.26), **(B)** relative humidity (%) (p-value = 0.000***), and **(C)** accumulated total precipitation (mm) (p-value = 0.99) on the abundance of points containing eggs of *S. latifascia*. The second model characterized the effects of **(D)** the mean temperature (°C) (p-value = 0.014*), **(E)** relative humidity (%) (p-value = 0.26), and **(F)** accumulated total precipitation (mm) (p-value = 0.73) on the abundance of *S. latifascia* moths.

**Figure 9:**
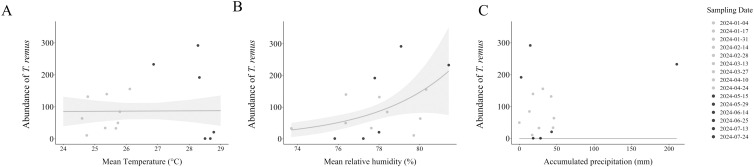
Effect of the local weather on Telenomus remus. The solid line illustrates the predicted values from the generalized linear model (GLM), and the shaded areas indicate 95% confidence intervals (CIs). The solid dots indicate the warmest time period and opaque dots the mildest time period as determined by temperature (°C) (**Supplementary Table S3**). The model characterized the effect of the **(A)** mean temperature (°C) (p-value = 0.97), **(B)** relative humidity (%) (p-value = 0.12), and **(C)** accumulated total precipitation (mm) (p-value = 0.85) on the abundance of *T. remus*.

The local weather observed during the field study influenced the abundance of points where the eggs were observed, as well as the abundance of moths (**Figure 8**). However, this same pattern is not evident for *T. remus* (**Figure 9**). Specifically, an increase in temperature did not affect the abundance of points with eggs (Estimate = -0.038, Std. Error = 0.048, *p*-value = 0.26). In contrast, the abundance of trapped moths was negatively affected by the rising temperatures (Estimate = -0.2, Std. Error = 0.081, *p*-value = 0.01^*^). Relative humidity had a significant effect on the abundance of points where the eggs were found (Estimate = 0.07, Std. Error = 0.02, *p*-value = 0.000^***^). Although it did not influence the abundance of trapped moths (Estimate = 0.061, Std. Error = 0.05, *p*-value = 0.26) and parasitoids (Estimate = 0.26, Std. Error = 0.17, *p*-value = 0.11).

## 4 Discussion

For the first time, an established population of *T. remus* was documented in Puerto Rico, specifically in a region characterized by extreme summer temperatures and prolonged periods of drought. Remarkably, all 1,443 specimens identified as parasitizing *S. latifascia* eggs belonged to this same species, suggesting a particular host-parasitoid interaction that persisted across time and space.

To understand the factors influencing their dynamics, we studied the attractiveness of two commercial sex pheromones used for noctuid species, as well as the effects of agricultural landscape metrics and local weather on the oviposition behavior and abundance of *S. latifascia* and *T. remus*. This research provided insights into natural conditions that, to our knowledge, have not been previously reported in the literature.

### 4.1 Dynamics of host-parasitoid and sex pheromone over time

Our first research question examined the overall attraction of host and parasitoid in relation to two types of sex pheromones over time. To investigate this, we observed a unique oviposition behavior of *S. latifascia* under natural conditions. Females of this species do not exhibit selectivity while searching for suitable hosts to lay their eggs (18). This behavior compromises the survival and developmental performance of the future offspring (19). Additionally, locating these egg masses among various potential hosts is time-consuming, as they could be easily overlooked on the plants due to their greenish color. These challenges may lead to underestimations and misinterpretations regarding their real dynamics. For these reasons, the attraction of *S. latifascia* females to lay eggs on plastic surfaces may be an important methodology for collecting eggs in the field. An important aspect of our methodology is that egg collection points remained fixed in the field over time.

Our results indicate that traps containing pheromone 2, which consists of (Z)-11-Hexadecenyl acetate, (Z)-7-Dodecenyl acetate, and (Z)-9-Tetradecenyl acetate, generally attracted more egg-laying females, trapped moths, and parasitoids. However, it is important to note that the only significant difference observed was in the presence of trapped moths. This pheromone blend was originally developed for *Spodopetra frugiperda* but can also attract non-target species of noctuid moths (17).

Interestingly, during our field research, females of *S. latifascia* were also attracted to the traps containing these formulated sex pheromones derived from female glands. This attraction might be related to the similarities in pheromone components used by closely related species, which often share the same compounds in different proportions, leading to overlapping blends (52, 53). Imperfect mate recognition may contribute to the attraction of *S. latifascia* to pheromone traps intended for *S. frugiperda*.

Regarding parasitoid presence, we found no statistically significant differences in parasitoid preference based on the type of pheromone. Notably, parasitoids in eggs attached to the traps added complexity to our study, raising questions about the cues used by *T. remus* females to locate hosts in natural environments. Egg parasitoids use multiple chemical cues simultaneously to locate their hosts, which is crucial in complex landscapes (54). The literature indicates that plants can release complex mixtures of volatile compounds in response to insect herbivory, indirectly guiding natural enemies to their hosts (21, 55). However, we observed eggs and parasitoids on traps surrounded solely by bare soil. Our findings suggest that *T. remus* may be guided by three potential clues: the sex pheromone used in the traps, semiochemicals released by female moths, or compounds released by egg masses. The semiochemicals released by the host represent a direct form of interaction with the parasitoid (56).

### 4.2 Parasitism rate of *Spodoptera latifascia* eggs

Our second aim was to assess the overall parasitism rate of *T. remus* on *S. latifascia* eggs, a dynamic that was never studied under field conditions. It is important to note that the parasitism reported in this study may be underestimated due to the use of non-natural substrate (plastic surfaces) for egg collection. Despite this limitation, the highest parasitism recorded within the sampling dates were on March 13 and May 29, with 33% of the total eggs collected parasitized. When analyzing individual points, one sampling location containing 122 eggs exhibited a 95% parasitism rate. In contrast, two other sampling points with 48 and 56 eggs each showed 100% parasitism rates. These findings underscore the effectiveness of this *T. remus* isoline and its potential as a natural pest control agent. Most studies on *T. remus* parasitism focus on *S. frugiperda* due to its worldwide importance (2, 57). These studies have shown natural parasitism of 60% for 103 eggs under field conditions (2). Under controlled conditions, parasitism rates can reach 80% on masses comprising approximately 150 eggs (57).

Egg masses of *S. latifascia* are highly aggregated, usually consisting of superposed layers that can range from one to four. This structural aggregation may pose significant challenges to parasitism. Our findings suggest that an increase in the number of eggs resulted in a decrease in *T. remus* emergence, indicating that the layered arrangement of egg masses may challenge parasitoid oviposition. The phenomenon of egg layering is a defense mechanism against natural enemies that has also been documented in other *Spodoptera* species (10, 12, 13, 58).

Additionally, we observed an interesting trend regarding the abundance of moths and parasitoids: as the number of trapped moths increased, the parasitism rate decreased. The growth in the population of *S. latifascia* moths may be associated with an increased number of egg masses in the system, which may correlate with a decline in the abundance of *T. remus* wasps. This phenomenon may be related to the upper threshold of parasitoid growth. When the host population exceeds the capacity of its parasitoid, parasitism reaches its limit. This pattern has already been reported in other species (59, 60). However, this relationship may also be influenced by multiple ecological factors beyond just host density alone.

### 4.3 Landscape structure and function

The third aim of this study was to evaluate the relationship between the landscape structure and function in host-parasitoid dynamics. Many studies on parasitism rates and parasitoid behaviors have been conducted under laboratory conditions, which may not accurately reflect traits observed in the field (54, 56, 61). Host-parasitoid dynamics, particularly in relation to landscape factors, are often overlooked, underscoring the need for more information on how plant communities influence both parasitoids and their hosts (54).

Regarding the structural landscape metrics, our findings indicate a positive relationship between the total number of patches, which represent an area distinguishable within the surrounding area, and the abundance of *T. remus*. Areas dominated by weeds were also positively associated with parasitoid abundance, although the analysis was not statistically significant.

We observed that the landscape structural metrics, such as the total number of patches associated with *T. remus* abundance, did not impact *S. latifascia*. Therefore, it is crucial to include additional vegetation traits in the analysis, specifically the availability of flowering plants, which can be described by the phenological stages of the plant community. We divided vegetation composition into two phenological stages, vegetative and reproductive. We also considered plant residues in our landscape functional analysis. When these functional traits were evaluated, we found that pumpkin residues negatively affected moth abundance. Meanwhile, the abundance of points with the presence of eggs was positively associated with cucumber during the reproductive stage. Regarding the abundance of *T. remus* in the functional landscape, we found that flowering weeds may provide an essential source of nutrients for adult parasitoids, thereby increasing the likelihood of egg parasitism in these patches.

Weeds contribute to functional diversity in the agricultural system. A more diverse plant composition enhances the longevity and effectiveness of natural enemies by providing food resources to parasitoids (20, 62, 63). Other studies have shown that weeds influence the dynamics of *T. remus* (64). While weeds are highly adapted to their environment and can support biological control during their flowering stage, they also negatively impact crops by competing for resources. Therefore, effective management strategies that integrate multiple disciplines, such as herbology and entomology, are necessary to optimize natural biological control and minimize the impact of weeds in agricultural systems (61).

### 4.4 Influence of weather on population dynamics

Our last aim was to evaluate the influence of weather variables on the abundance of eggs of *S. latifascia* moths and *T. remus* parasitoids. We found that increased temperatures affected the abundance of moths. Unfortunately, there is limited research on temperature thresholds and other weather-related variables affecting *S. latifascia*. Studies on different species of the genus *Spodoptera*, such as *S. frugiperda*, have indicated that optimal development occurs at temperatures between 26°C and 30°C and that higher temperatures decrease the egg-to-adult development time under laboratory conditions (65). On the other hand, *S. eridania* does not tolerate temperatures above 34°C, while *S. albula* can survive in warmer regions (66). These findings highlight considerable variability within the genus *Spodoptera* and a notable lack of information on how these species behave in tropical regions. We did not observe a clear relation between temperature variation and the abundance of *T. remus*. Previous studies have indicated that the parasitism behavior of *T. remus* under laboratory conditions varies with temperature and is influenced by the *Spodoptera* species. For example, Pomari et al. (13) found that the optimal temperature for *T. remus* was between 22°C and 25°C, with a significant decrease in emergence at 34°C.

Our analysis revealed that humidity significantly affected the occurrence of *S. latifascia* eggs. Humidity prevents egg desiccation, ensuring the successful emergence of neonates or parasitoids. Higher humidity levels have also been linked to improved biological performance of parasitoids (67).

## 5 Conclusion

This research represents the first report of *T. remus* in Puerto Rico. Our study presents new insights into the factors affecting the population dynamics of *S. latifascia* and *T. remus* in the field. We utilized a method to assess the parasitism rate that has not been previously documented in the literature, providing innovative approaches to enhance egg collection in the field. Our findings emphasize the complexity of host-parasitoid behavioral responses to natural conditions. We observed that only the presence of *S. latifascia* was influenced by the type of pheromone used in the traps. In contrast, the presence of eggs and parasitoids remained consistent regardless of pheromone types. Furthermore, the landscape factors affecting *S. latifascia* differ from those influencing *T. remus*. However, *T. remus* demonstrated considerable efficacy in host searching, underscoring its importance for augmentative biological control initiatives. Moreover, our analysis of local weather parameters revealed that humidity is a pivotal factor influencing the population dynamics of both species.

## Data Availability

*Telenomus remus* sequences data have been deposited in the NCBI database under the accession numbers PV946711 to PV946723. Voucher specimens used for the molecular analyses were deposited in the Oscar Monte Entomophagous Insect Collection in the Instituto Biológico, Campinas, São Paulo, Brazil (IB-CBE 008802 to IB-CBE 008821). Specimens were also deposited in the National Museum of Natural History, Smithsonian Institution (USNM – 2097404, USNMENT01916843-USNMENT01916859), and in the Museo de Entomología y Biodiversidad Tropical of the University of Puerto Rico (MEBT-l 00488664). The field data that support our findings in this study are available upon request to the corresponding authors.
