# Supplementary material for: First report of natural parasitism by Telenomus remus Nixon, 1937 on Spodoptera latifascia Walker, 1856 eggs in Puerto Rico: insights into spatial-temporal dynamics in a semiarid tropical region
Source: Front Insect Sci. 2026 Jan 26;5:1727464. doi: 10.3389/finsc.2025.1727464 (PMC12884642; doi:10.3389/finsc.2025.1727464)
Supplement: Supplementary file 1 [file DataSheet1.docx]

Supplementary Material

# Supplementary Video

Spatial and temporal distribution of functional land cover metrics, which categorize land cover based on two phenological stages: vegetative and reproductive, to indicate the abundance of flowers. Black dots represented the locations where *Spodoptera latifascia* eggs were present, while pink dots indicate areas where parasitoids have been observed. The size of the pink dots reflects changes in parasitoid abundance, with larger dots signifying higher abundance.

**
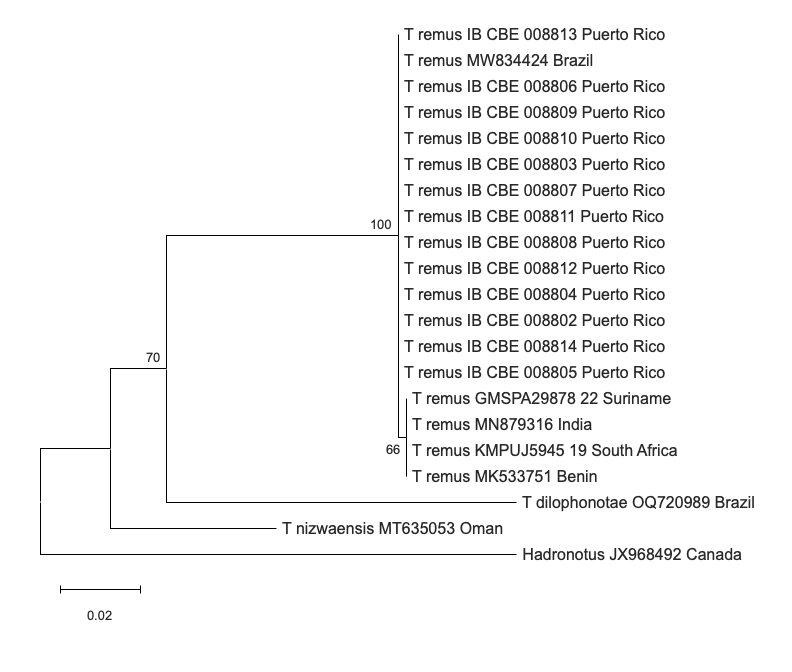
**

**Figure S1.** Phylogenetic tree showing the clustering of associated taxa. The numbers at the nodes represent the percentage of replicate trees where these taxa are grouped.

**Table S1.** Mean parasitism rates, and the proportion of males and females.

| **Date** | **Sex Ratio** | **Proportion of Males** | **Proportion of Females** |
| --- | --- | --- | --- |
| 01/04/2024 | 0.83 | 0.45 | 0.55 |
| 01/17/2024 | 0.96 | 0.49 | 0.51 |
| 01/31/2024 | 1.08 | 0.52 | 0.48 |
| 02/14/2024 | 0.57 | 0.36 | 0.64 |
| 02/28/2024 | 2.63 | 0.72 | 0.28 |
| 03/13/2024 | 0.66 | 0.40 | 0.60 |
| 03/27/2024 | 39.50 | 0.98 | 0.02 |
| 04/10/2024 | 0.23 | 0.19 | 0.81 |
| 04/24/2024 | 0.79 | 0.44 | 0.56 |
| 05/15/2024 | 1.15 | 0.53 | 0.47 |
| 05/29/2024 | 0.79 | 0.44 | 0.56 |
| 06/14/2024 | 0.91 | 0.48 | 0.52 |
| 06/25/2024 | 0.56 | 0.36 | 0.64 |

**Table S2**. presents a Redundancy Analysis (RDA) illustrating the correlations between various structural and functional landscape metrics and host-parasitoid dynamics. This analysis includes the abundance of points where eggs were located, the population dynamics of the moth species *Spodoptera latifascia*, and the abundance of the parasitoid *Telenomus remus*.

| **Landscape metrics** | **Variables** | **df** | **Variance** | **F** | **Pr(>F)** |
| --- | --- | --- | --- | --- | --- |
| Structural | Patches total | 1 | 0.070 | 12.52 | 0.008^**^ |
|  | Cucumber | 1 | 0.020 | 3.557 | 0.097 . |
|  | Onion | 1 | 0.002 | 0.425 | 0.544 |
|  | Pumpkin | 1 | 0.001 | 0.316 | 0.650 |
|  | Tomato | 1 | 0.002 | 0.039 | 0.576 |
|  | Watermelon | 1 | 0.000 | 0.011 | 0.853 |
|  | Weeds | 1 | 0.021 | 3.764 | 0.082 . |
|  | Residual | 7 | 0.039 |  | |
| Functional |  |  |  |  |  |
|  | Patches total | 1 | 0.031 | 7.6897 | 0.056 . |
|  | Flowering vegetative | 1 | 0.001 | 0.375 | 0.651 |
|  | Flowering cucumber | 1 | 0.004 | 1.200 | 0.338 |
|  | Onion residue | 1 | 0.006 | 1.53 | 0.291 |
|  | Plantain | 1 | 0.007 | 0.185 | 0.740 |
|  | Plantain Residue | 1 | 0.015 | 3.765 | 0.132 |
|  | Pumpkin vegetative | 1 | 0.003 | 0.972 | 0.406 |
|  | Flowering pumpkin | 1 | 0.000 | 0.098 | 0.853 |
|  | Pumpkin residue | 1 | 0.026 | 6.612 | 0.069 |
|  | Flowering watermelon | 1 | 0.002 | 0.563 | 0.550 |
|  | Flowering weeds | 1 | 0.002 | 0.563 | 0.550 . |
|  | Residual | 3 | 0.0121 |  | |

Note: Significant codes: 0’^***^ ‘ 0.001 ‘^**^ ‘ 0.01 ‘^*^ ‘ 0.05 ‘.’ 0.1 ‘ .‘.

**Table S3**. Sampling dates and the classification of periods based on local temperature. Time period 1 represents the warmest period, while time period 2 corresponds to the coldest months of the year.

| **Sampling number:**  **eggs and moths** | **Sampling number: Parasitoids** | **Date** | **Time Period** |
| --- | --- | --- | --- |
| 1 | - | 07/05/2023 | 1 |
| 2 | - | 07/19/2023 | 1 |
| 3 | - | 07/30/2023 | 1 |
| 4 | - | 08/16/2023 | 1 |
| 5 | - | 08/31/2023 | 1 |
| 6 | - | 09/13/2023 | 1 |
| 7 | - | 09/25/2023 | 1 |
| 8 | - | 10/11/2023 | 1 |
| 9 | - | 10/25/2023 | 1 |
| 10 | - | 11/08/2023 | 1 |
| 11 | - | 11/21/2023 | 1 |
| 12 | - | 12/06/2023 | 2 |
| 13 | - | 12/18/2023 | 2 |
| 14 | 1 | 01/04/2024 | 2 |
| 15 | 2 | 01/17/2024 | 2 |
| 16 | 3 | 01/31/2024 | 2 |
| 17 | 4 | 02/14/2024 | 2 |
| 18 | 5 | 02/28/2024 | 2 |
| 19 | 6 | 03/13/2024 | 2 |
| 20 | 7 | 03/27/2024 | 2 |
| 21 | 8 | 04/10/2024 | 2 |
| 22 | 9 | 04/24/2024 | 2 |
| 23 | 10 | 05/15/2024 | 1 |
| 24 | 11 | 05/29/2024 | 1 |
| 25 | 12 | 06/14/2024 | 1 |
| 26 | 13 | 06/25/2024 | 1 |
| 27 | 14 | 07/13/2024 | 1 |
| 28 | 15 | 07/24/2024 | 1 |
